# Alcohol—Its Theraputic Action and Remedial Agency

**Published:** 1875-03

**Authors:** Jas. S. Whitmire

**Affiliations:** Metamora, Ill.


ARTICLE II.

### ALCOHOL — ITS THERAPEUTIC ACTION AND REMEDIAL AGENCY.

Read before the Woodford County Medical Society, at its Semi-Annual Meeting, at Minonk, January 5th, 1875.

BY JAS. S. WHITMIRE, M.D., METAMORA, ILL.

*Mr. President, and Gentlemen of the Society:* At our last Annual Meeting, at El Paso, I was designated, by our President, to submit a report at this meeting upon the therapeutic and remedial agency of alcohol in the treatment of disease. I cannot promise you that this much-abused article—as a beverage, by its recipients, and, also, abused on account of its use by another class, who conceive it to be the refinement of abomination, on the one hand; and on the other, by a different class and for greatly different reasons, laud its virtues to the skies as a therapeutic and remedial agent—will receive anything like a complete and critical examination, in all its bearings in regard to its effects on the economy by me, because our ablest therapeutists and most learned physiologists and pathologists have not, as yet, fairly settled the question as to whether the action of alcohol, in any of its various forms, is a heater or a cooler upon the animal organism. In other words, whether it is a promoter of life force by its oxydation in the blood and tissues, or, whether its ultimate effects are not to produce enervation by the direct poisoning of the blood and tissues of the body, the obstruction of molecular changes, and lessening the natural secretions of the glandular system. In regard to the mooted question, with respect to its pernicious or other effects upon the healthy animal organism, when its use is abused, or even when temperately used, I am not called upon in this connection to either advocate or condemn, because there will always be an abundance of controversy among enthusiasts, pro and con, in regard to it. I am called upon, however, i this place to inquire into its therapeutic action and curative agency in disease, and while my investigations may compel me to treat somewhat of its physiological action on the economy in disease, it is more than probable that my conclusions may be neither agreeable nor satisfactory to either of the exclusive factions.

There are two theories in regard to the therapeutic action of alcohol—one, that it is a direct and positive stimulant, and the other, that it is a sedative by producing paresis, or want of sensibility, in the vaso motor system. In regard to these, they may both be right in one sense, and both wrong in another, according to circumstances, and because of the conditions under which it may be administered. The stimulant theory is supported on the ground that it gives increased force and frequency to the heart's action; that additional energy is transmitted to the muscular system, and that it produces temporary exaltation in the mental faculties. On the other hand, it is claimed, notwithstanding the effects mentioned as favoring the stimulant theory, that these apparent effects may be accounted for on different and more rational grounds than that of direct stimulation, and these are, that alcohol possesses a food-producing force, and indirectly nutritious qualities, which, by promoting digestion and sanquification, prompts a more thorough appropriation of food to nutrition; and that the saving thus effected to the general system, more than counterbalances the waste of the tissues which is implied by increased vital action. From the direct effect of alcohol upon the general system, and especially that of the nervous functions, there is produced a paresis of the capillary vessels, and more especially of the cutaneous surface, which is propagated specially through the vaso motor tract. On account of this want of excitability of the capillaries, they become abnormally filled with blood, which is only relieved by a great increase of cutaneous transpiration, and also by increased exhalation from the lungs; these both being cooling processes, the temperature of the body is kept in its normal condition; hence the apparently stimulating effect produced by liberal—but not too abundant—potations of alcohol are entirely counterbalanced through and by natural chemico-physiological law.

Alcohol is considered by Dr. Pavy, of London, who has written a late work on Dietetics, as intermediate, as food, for the production of *life force*, between the hydrocarbons, or fats, and the carbo-hydrates, as starch and sugar. He also accepts the demonstrations made by Anstie, Dupre and others, which establish the fact that ingested alcohol is practically and wholly destroyed within the system, hence, his obvious conclusions are, that if this be the case, it may be fairly assumed that the destruction of alcohol in the system is attended with oxydation, and hence, a corresponding liberation of force, unless, indeed, it should undergo some peculiar change or metamorphosis into a principle to be temporarily retained, but, nevertheless, ultimately to be called upon by the requirements of the system and applied in *force* production.

In this day of scientific research, investigation and progress, it will not do to assume a certain state of facts to be true, and then because a certain class of conditions exist, go to work and construct a theory from these conditions that goes to show or support the truth of the original assumption. That day has passed away with many unfounded theories of the past, and a new era has been ushered in, where everything, material and apparently immaterial, must be placed in the chemist's crucible, and subjected to the heat of intellectual fire, where it is purified, and separated into its ultimate elements and original constituents by the magnetic battery of reason, before a fact is established, upon the basis of which we may establish axioms. When once our axioms are constructed by such process, we then have unqualified data from which we may deduce our theories, and from which rational deductions must necessarily result.

Hence, in our day the therapeutic and physiological action of alcohol upon the animal organism, either in health or disease, is no longer hypothetical, but its mode and manner of action is a fact fixed and proven to exist, not only by philosophical experiment, but also by its chemico-physiological relations to vital force. Experience had long since taught the early members of our profession that alcohol, in its various forms, was not only a certain but a powerful remedial agent in the treatment of the low grades of fever, and also, in acute diseases where the lamp of life was barely flickering with a sickly flame, when nervous prostration and want of vital force was the principal impediment to recovery; but it was not till within the last decade that chemico-physiological research has demonstrated the true position that it occupies as a remedial and restorative agent, by the minute and searching investigation of its chemical affinities in the living organism and the relations that it holds to other ingesta that is common for the nutrition of man. Within this very brief period, we have demonstrated the why, the wherefore, and the very nature of its action on the economy in both health and disease, and had pointed out to us the various conditions that are appropriate for its remedial adminstration; and hence, we may now prescribe it with an almost mathematical precision and certainty as to its ultimate results; therefore we no longer grope our way in the dark as those who preceded us were compelled to do, administering alcohol and other drugs simply and purely upon theoretical grounds. Now, when we see our patients lingering for the want of vital force; when we find them dull and stupid and in a condition of low muttering delirium, with subsultus tendinum from inanition, or nervous exhaustion, it matters not what the cause of the prostration may have been, we know from the condition present, and in connection with physical diagnosis, that our patient needs nourishment in connection with alcohol in some of its forms, in order that the latent fires pent up in the organism may be made to burn and rehabilitate the vital forces. He needs food for the muscular tissue to supply the waste ; food for the cellular and connective tissues to supply their decay ; food for the osseous or bony structure, to rebuild its wasting strength ; and phosphorized food for the nervous system to rekindle its rapidly waning magnetic fire and restore its wonted functions. In the condition that we now find our patients, we know what they need, and the thing to be accomplished is, to see that the necessary nourishment for all these tissues is provided in abundance, and properly prepared for such digestion as the stomach is capable of performing, and made ready for assimilation. When this is once accomplished, the tissues of the body, which have been undergoing continual waste and disintegration, will be reconstructed by the appropriation of this food to the several tissues, according to their wants. The breaking down and building up of these different structures, you are aware, are accomplished by vitalized molecular change and interstitial development of new cell structure. These things must all be accomplished through and by the integrity of the vital forces, and these forces must be kept alive by ingesta that contain a heat-producing force or power. It is then apparent that some of the alcoholic beverages, along with the simplest character of nitrogenized food that contains principles that are the most easily oxydized, should comprise our selections for this purpose ; because, in the chemico-vital union of that element (oxygen) with all the carbonaceous and effete matters of the system, from whatever source they may have been derived, heat, and consequently vital force, is evolved, and this is taking place in every organ and tissue of the body continually, in the living organism. Therefore, it is through this vivifying, life-giving and life-sustaining principle (oxygen) that the rudimentary construction cells are formed, and that are continually forming in every structure, organ and tissue of the body and preparing to take the place, by assimilation, of those parts that have served their purpose, broken down and been carried out of the economy as of no further use.

From the careful study and investigation of the foregoing facts, in all their rational bearings, it may be seen that invalids and aged persons who contain a large amount of latent or undeveloped force or power, may be greatly benefited by the moderate use of some one or another of the milder alcoholic beverages ; because, as we stated in the beginning of this paper, these beverages, used in proper moderation, and under appropriate restrictions, facilitate digestion by their temporary stimulation, and remotely, they furnish some nutriment upon which vitality feeds, and by its own destruction by oxydation facilitates sanguification, and thus causing a more thorough appropriation of food to nutrition. Therefore, these invalids and aged persons, by the use of these beverages, will have their nutrition increased, their warmth promoted, and consequently their latent force or power developed and brought into action, and their moving, working, active power, whether it be mental or physical power, greatly augmented. This being the case, —I may say the facts in the premises, —who is there in these days of enlightened reason, who is bold enough to discard this powerful restorative agent, (alcohol in its various dilute forms), in the promotion of health and longevity, from our catalogue of medicaments, or even from our list of nutritive dietetics and restoratives? Enthusiasts may prescribe its use under any circumstances ; zealots may consign it to the halls of pandemonium ; but enlightened reason will always pronounce a benediction upon it.

It is argued as against the use of alcohol as a medical agent, that it is apt to create an unnatural appetite in the recipient for the habitual and unnecessary use of the article, and that in such case there will result a lasting and untold evil to the individual ; and besides, it would, by example, spread a baleful influence in the society where it was used. This may be all true as a matter of fact, but I apprehend that in 99 per cent. of all the individuals who abuse the use of alcoholic liquors, they have learned to crave it from a habit contracted from other sources than that of the educated physician. That the habitual use of alcohol, when it is not absolutely needed to accomplish some needed purpose in the economy, is detrimental to health, there can be no doubt; but that it is so certain to create an abnormal and insatiable appetite that it will lead to its abuse, is more problemetical — it is simply hypothetical; and for this reason we cannot afford to give up a positive and substantial good for fear of an imaginary evil.

In this connection we have only been called upon to inquire into the remedial agency of alcohol in disease, and the conditions under which it exerts its most favorable influence as a therapeutic agent, and to illustrate the most probable theory of its action as a force producing power. This we have endeavored to do to the best of our ability, and we are satisfied with the conclusions to which we have arrived. And in this connection we will simply remark that the evil effects that result from the abuse of any powerful agent can never be allowed as an argument against its use; but the evils produced by the abuse of alcoholic beverages are incalculable—they are beyond computation, and their influence is felt for the worse in the degradation of individuals in every community. Against this evil we can only point out its dangers by pointing to the utter ruin produced by their indiscriminate and unnecessary use; and against its use we may encourage philanthropy and morality to continue their crusade even to the extent of the utter abolition of its use, excepting by and with the advice of a thoroughly competent, educated and conscientious physician.

